# *Anopheles arabiensis* egg treatment with dieldrin for sex separation leaves residues in male adult mosquitoes that can bioaccumulate in goldfish (*Carassius auratus auratus*)

**DOI:** 10.1002/etc.2371

**Published:** 2013-10-21

**Authors:** Hanano Yamada, Zora Jandric, Sorivan Chhem-Kieth, Marc JB Vreysen, Mohammad N Rathor, Jeremie RL Gilles, Andrew Cannavan

**Affiliations:** 1 Insect Pest Control Laboratory, Joint Food and Agriculture Organization/International Atomic Energy Agency Division of Nuclear Techniques in Food and Agriculture, International Atomic Energy AgencyVienna, Austria; 2 Food and Environmental Protection Laboratory, Joint Food and Agriculture Organization/International Atomic Energy Agency Division of Nuclear Techniques in Food and Agriculture, International Atomic Energy AgencyVienna, Austria; 3 Department of Life Sciences, University of the West IndiesSt. Augustine, Trinidad

**Keywords:** Dieldrin, Bioaccumulation, Sterile insect technique, Gas chromatography

## Abstract

The sterile insect technique (SIT) is a biological control tactic that is used as a component of area-wide integrated pest management (AW-IPM) programs. The SIT can only be applied against disease-transmitting mosquitoes when only sterile male mosquitoes are released, and the blood-sucking and potentially disease-transmitting females are eliminated from the production line. For *Anopheles arabiensis*, a potent vector of malaria, a genetic sexing strain was developed whereby females can be eliminated by treating the eggs or larvae with the insecticide dieldrin. To evaluate the presence of dieldrin residues in male mosquitoes designated for SIT releases, a simple, sensitive, and accurate gas chromatography–electron capture detector (GC–ECD) method was developed. In addition, bioaccumulation and food chain transfer of these residues to fish after feeding with treated mosquitoes was demonstrated. The overall recovery from method validation studies was 77.3 ± 2.2% (mean ± relative standard deviation [RSD]) for the mosquitoes, and 99.1 ± 4.4% (mean ± RSD) for the fish. The average dieldrin concentration found in adult male *An. arabiensis* was 28.1 ± 2.9 µg/kg (mean ± standard deviation [SD]). A range of 23.9 ± 1.1 µg/kg to 73.9 ± 5.2 µg/kg (mean ± SD) of dieldrin was found in the fish samples. These findings indicate the need to reassess the environmental and health implications of control operations with a SIT component against *An. arabiensis* that involves using persistent organochlorines in the sexing process.

## Introduction

The mosquito *Anopheles arabiensis* is an important vector of malaria, of which there were 216 million cases in 2010, causing 655 000 deaths in 106 endemic countries. 1In addition to significant efforts to control the disease using insecticides, bed nets, repellents, and environmental control tactics, efforts have been revived to develop and validate sterile insect techniques (SIT) [2,3] as an additional control tactic for use in area-wide integrated pest management (AW-IPM) programs [4]. The SIT requires mass-rearing of the target species in large production centers and releasing sterile (male) insects in overflooding numbers [5] with the aim of suppressing the wild population.

One of the many essential requirements of applying the SIT for mosquitoes is the release of only sterile males, which requires eliminating female mosquitoes from the production line. Female mosquitoes, even when sterilized with ionizing radiation, cannot be released, because they retain their vectorial capacity. Because manually separating the sexes based on their morphology is highly impractical, potentially inaccurate, and time- and laborintensive, genetic sexing strains (GSS) based on an artificially induced sex linkage of a selectable allele are required [6]. A GSS of *An. Arabiensis*, ANO IPCL1, based on a mutation that confers resistance to dieldrin [7] was recently created. When treated with dieldrin, the susceptible females are killed, and the resistant males survive. Larval exposure for 1 h to dieldrin at 100 µg/L eliminates the susceptible females, whereas exposure of eggs to 2000 µg/L, 3000 µg/L, and 4000 µg/L dieldrin solutions also results in complete female elimination [7] without significantly reducing the number of males produced.

Dieldrin (C_12_H_8_Cl_6_O) is considered a persistent organic pollutant that does not readily breakdown and remains stable in soil and in ultraviolet light [8]. Furthermore, it tends to biomagnify in the food chain [9]–[11], where it is stored mostly in the adipose tissue of insects and mammals [12,13]. The main concern with dieldrin arose after many years of indiscriminate applications over large areas, when levels of insecticide residues above acceptable thresholds were detected in dairy milk, cheese, and adipose tissue of birds, cows, and even humans [14]. This led to its ban by the US Environmental Protection Agency (USEPA) by 1980. In the 1970s and 1980s, some isolated experiments were conducted in response to these concerns, in which bioaccumulation or food chain transfer was detected in the laboratory [9,10,12,15] or in the environment [16,17]. These studies involved relatively high doses of the insecticide, however, which were artificially fed to the experimental animals or sprayed in the environment over prolonged periods of time. Because the concentrations of dieldrin currently used in the sex separation process of *An. arabiensis* are no higher than 2000 µg/kg when treating mosquito eggs, or 100 µg/kg when treating larvae, the hypothesis of the present study was that residues retained by the adult mosquito would be negligible, with no concern for biomagnification and accumulation of the insecticide in the environment and food chain. To test this hypothesis, experiments were conducted to determine the level of dieldrin residues in adult male *An. arabiensis* that were treated at the egg stage with solutions of 2000 µg/kg of dieldrin and whether these residues in the mosquitoes were accumulated and magnified in fish that were fed these mosquitoes over prolonged periods of time. The results should resolve or confirm any concern about implementing SIT programs to control *An. arabiensis*, whereby large numbers of dieldrin-treated mosquitoes are to be released into the environment.

Gas chromatography (GC) equipped with an electron capture detector (ECD) is used routinely to analyze organochlorine pesticides in fish [18]. Various clean-up methods have been reported to remove co-extracted fish lipids, including partitioning with acetonitrile and petroleum ether followed by a florisil column to remove residual oil [19]; gel permeation chromatography, sweep co-distillation, and florisil column adsorption chromatography [20]; and solid phase extraction [21]. Determining dieldrin in mosquitoes has been reported by GC–ECD using acetone-based extraction [22].

The main objectives of the present study were to quantify the residues of dieldrin retained by treated mosquitoes, to assess indicators for bioaccumulation, and to evaluate the possibility of food chain contamination indicated in aquatic animals present in treated insect release areas. The research thus consisted of 3 parts: setting up an experimental model using dieldrin-treated mosquitoes that were subsequently fed to fish; extracting and quantifying dieldrin residues recovered from adult mosquitoes; and detecting dieldrin accumulated in fish after ingesting dieldrin-treated mosquitoes for a period of 6 mo as an indicator for bioaccumulation and food-chain transfer.

## Materials and Methods

### Chemicals and reagents

Dieldrin (≥90%), acetonitrile and toluene (high-performance liquid chromatography grade), and analytical grade magnesium sulphate (≥98.0%), sodium citrate tribasic dihydrate (≥99.5%), and sodium citrate dibasic sesquihydrate (≥99.0%) were provided by Sigma-Aldrich. Sodium chloride (≥99.5%) was supplied by Merck. The clean-up salts, Bondesil primary–secondary amine, and matrix solid phase dispersion C18 materials were obtained from Varian and Biotage (Uppsala, Sweden), respectively. Ultrapure water was prepared using a Milli-Q system (Millipore).

The concentrations of dieldrin residues were investigated in both mosquitoes and fish to assess the extent of the bioaccumulation process following the consumption of treated mosquitoes by fish. The samples were analyzed by GC–ECD, following extraction with acetone for mosquitoes and by a dispersive solid-phase extraction with acetonitrile for fish.

### Mosquitoes

Mosquitoes from the ANO IPCL1 genetic sexing strain of *An. Arabiensis* were obtained from the Food and Agriculture Organization/International Atomic Energy Agency's Insect Pest Control Laboratory, where they had been exposed to dieldrin as eggs for sexing purposes as described by Yamada et al. [7]. The eggs of ANO IPCL1 were treated in dieldrin solutions at 2000 µg/kg for up to 24 h. These were then rinsed thoroughly and returned to rearing trays with deionized and larval food and were reared to adulthood. Following their deaths (after approximately 2 wk), the mosquito samples were frozen and freeze-dried.

The dieldrin content in treated mosquitoes was determined by acetone-based extraction. The mosquitoes were placed in glass vials in aliquots of 50 mg, then crushed and thoroughly mixed with 5 mL of acetone. The samples were then centrifuged (1360 *g*, 5 min, 15 °C) to induce clear separation of the acetone and the solid matrix. The supernatants (4.2 mL) obtained were dried under a gentle nitrogen stream, and the products redissolved in 0.5 mL of 15% acetone in isooctane. After filtration, the extracted products were transferred to GC vials for subsequent analyses by GC–ECD.

### Fish

Four fish *(Carassius auratus auratus)* were reared and kept according to Austrian animal care and protection regulations. The fish were given 3 mo to acclimate to the mosquito (untreated) diet and the environment, followed by a period of 6 mo, during which their diet consisted primarily of mosquito pupae and adults that had been treated with dieldrin as eggs. When insect material was in short supply, the fish were fed standard fish food pellets (Novo Pearl). On average, the fish each consumed 5 to 7 mosquitoes per weekday. They were not fed on weekends. The fish were kept in a static aquarium, supplied with aquatic plants. The water was filter-cleaned and aerated continuously and changed once every 4 wk. The fish were then given 4 mo to expel any un-sequestered dieldrin so that only permanently absorbed residues would be measured, before they were euthanized at 13 mo according to the Austrian animal protection and slaughter regulations.

The fish were decapitated and descaled, then individually thoroughly homogenized. The samples were stored at −20 °C for subsequent sample preparation and GC–ECD analysis. The 4 fish were recorded as number 1, 2, 3, and 4 and were all of similar weight (10–12.5 g) at the start of the experiment. At the time of euthanasia, their respective weights were 17.0 g, 21.4 g, 48.1 g, and 66.9 g. The variation in weight was due to the different growth rates of the individual fish over the 13 mo, during which some fish may have consumed more of the plants available in the aquarium than did the others. Determination of dieldrin in the treated fish was achieved using acetonitrile and dispersive solid-phase extraction. Samples (1 g) were weighed into polypropylene centrifuge tubes. Liquid–liquid partitioning was conducted by successively adding 5 mL water, shaking by hand (30 s), then adding 5 mL acetonitrile and shaking again (30 s). A mixture of magnesium sulphate (MgSO_4_; 2 g), sodium chloride (NaCl; 0.5 g), sodium citrate tribasic dihydrate (C_6_H_5_Na_3_O_7_x2H_2_O; 0.5 g), and sodium hydrogen citrate sesquihydrate (C_6_H_6_Na_2_O_7_x1.5H_2_O; 0.25 g) was added. The samples were then shaken by hand (1 min) and centrifuged (3180 *g*, 5 min, 15 °C). The sample extract aliquots (4 mL) were cleaned by adding a mixture of materials comprising MgSO_4_ (150 mg), primary–secondary amine (50 mg), and matrix solid phase dispersion C18 (50 mg) per mL of aliquot used. After vigorous vortex-mixing (30 s) and centrifugation of the tubes (1364 *g*, 5 min, 15 °C), 2 mL of the supernatants was transferred to glass tubes and evaporated using a TurboVap LV nitrogen evaporator (Zymark). The samples were evaporated to dryness, redissolved in 0.5 mL of toluene, and vortex-mixed (1 min). After sonication for 5 min in an ultrasonic bath (Model USC 300T; VWR), the samples were filtered through 0.45 µm polytetrafluoroethylene syringe filters, into GC vials and analyzed by GC–ECD.

### Dieldrin standard solutions

The dieldrin stock standard solution (1 g/L) was prepared in toluene. From the dieldrin stock standard solution, intermediate and working standard solutions were prepared (100 ng/µL, 1 ng/µL, and 0.1 ng/µL) with toluene and 15% acetone in isooctane as the solvent. Calibrators were prepared in blank matrix over the range 24 µg/kg to 480 µg/kg for mosquitoes and 2.5 µg/kg to 60 µg/kg for fish.

### Instrumental conditions

The chromatography was carried out using a Hewlett Packard (Agilent) Gas Chromatogram 6890 Series equipped with an HP-5 column (30 m × 250 µm inner diameter, 0.25 µm; Agilent) coupled with an electron-capture detector (temperature at 170 °C) in split mode. The analysis was performed with helium as the carrier gas at a flow rate of 1.5 mL/min; the initial oven temperature was 70 °C, with progressive heating to 300 °C (70 °C after 1.50 min, 110 °C after 2.50 min, 260 °C after 17.75 min, 300 °C after 22.75 min) with a total run time of 22.75 min. Data analysis was performed with the Agilent GC Chemstation (B.03.02) software and Microsoft Excel 2010.

### Method validation

#### Mosquitoes

Mosquito samples were fortified at 3 different concentrations (24 µg/kg, 120 µg/kg, and 240 µg/kg) with a dieldrin standard solution (100 ng/mL) prepared in 15% acetone in isooctane and analyzed using the method described above in the *Materials and methods*, Mosquitoes section. The full validation procedure was carried out on 3 d. Three replicates were prepared for each fortification level, and 2 different persons conducted the experiment.

#### Fish

Blank samples of *Carassius auratus auratus* were obtained from a pet store. These fish were not fed with contaminated mosquitoes. Samples were analyzed by the described method, and no dieldrin was detected (less than the limit of detection of the method). As with the adult mosquitoes, the method validation was conducted on 3 d by 2 different persons alternately. The samples were fortified at 3 concentrations (2.5 µg/kg, 12.5 µg/kg, and 25.0 µg/kg) with a dieldrin standard solution (100 ng/mL) prepared in toluene.

## Results

### Analytical method for dieldrin

#### Mosquitoes

The overall recovery yields for dieldrin in mosquitoes, using the validation protocol described above in the *Materials and methods, Method validation* section, averaged 77.3 ± 2.2% (mean ± relative standard deviation [RSD]) (Table1). The limit of detection and the limit of quantification were 7.5 µg/kg and 24.0 µg/kg, respectively.

**Table 1:** Overview of dieldrin average recoveries (R_A_; %) on 3 validation days, at 3 fortification levels (24 µg/kg, 120 µg/kg, and 240 µg/kg) and relative standard deviation (RSD; %) of male adult *Anopheles arabiensis* samples (*n* = 3)

Dieldrin spiking level (µg/kg)	Day 1		Day 2		Day 3		Overall days 1–3		Overall Levels	
	R_A_	RSD	R_A_	RSD	R_A_	RSD	R_A_	RSD	R_A_	RSD
24	74.8	6.1	78.2	3.7	78.3	4.0	77.1	2.6		
120	80.7	8.3	78.2	0.7	78.3	2.5	79.1	1.8	77.3	2.2
240	76.1	3.3	75.7	3.3	75.2	3.0	75.7	0.6		

#### Fish

**Figure 1:**
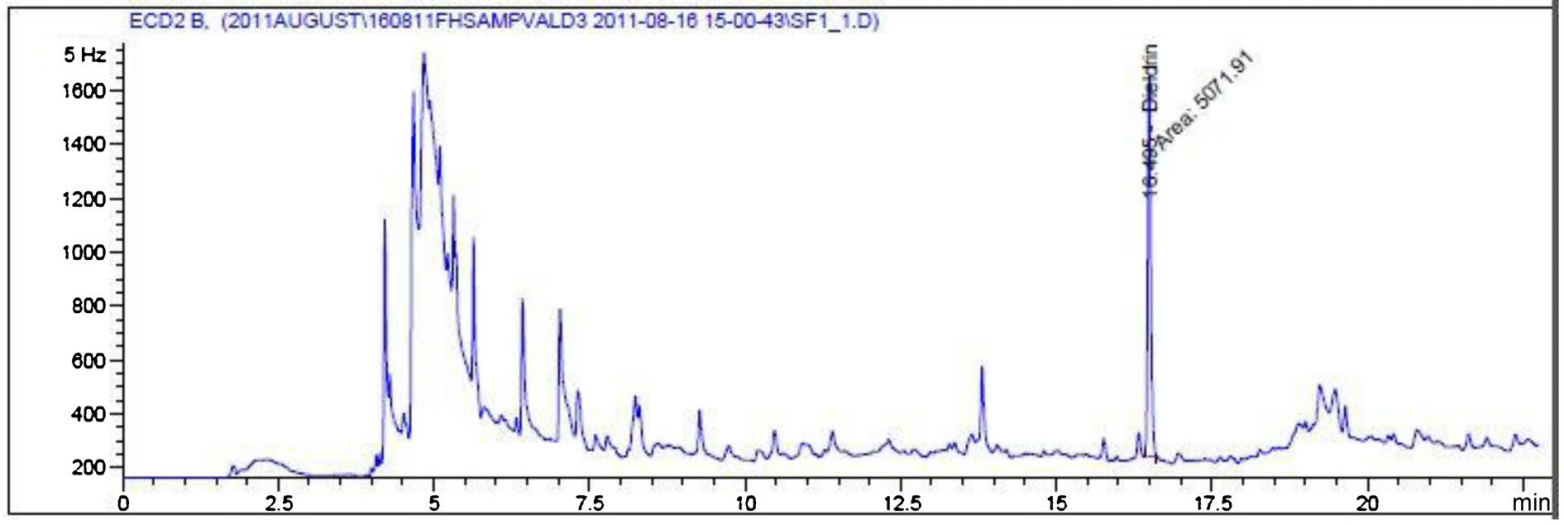
Dieldrin in a spiked fish sample at a concentration of 25.5 µg/kg.

The overall recovery yields for dieldrin in fish averaged 99.1 ± 4.4% (mean ± RSD) (Table2). The limit of detection and limit of quantification were 0.7 µg/kg and 2.5 µg/kg, respectively. A typical chromatogram of dieldrin, with a retention time of 16.5 min, in spiked fish samples at a concentration of 25.5 µg/kg is shown in Figure 1.

**Table 2:** Overview of dieldrin average recoveries (R_A_; %) on 3 validation days, at 3 fortification levels (2.5 µg/kg, 12.5 µg/kg, and 25 µg/kg) and relative standard deviation (RSD; %) of fish *Carassius auratus auratus* samples (*n* = 5)

Dieldrin spiking level (µg/kg)	Day 1		Day 2		Day 3		Overall days 1–3		Overall Levels	
	R_A_	RSD	R_A_	RSD	R_A_	RSD	R_A_	RSD	R_A_	RSD
2.5	105.8	4.9	95.7	5.2	87.9	6.6	96.5	5.6		
12.5	102.2	3.4	107.8	4.9	95.0	2.1	101.7	3.5	99.1	4.4
25	97.8	5.2	101.2	3.7	98.8	3.9	99.3	4.2		

**Table 3:** Dieldrin concentration in *Carassius auratus auratus* samples fed with *Anopheles arabiensis* that were treated with dieldrin as eggs

Sample name	Dieldrin concentration (µg/kg)	Standard deviation
Fish 1	67.4	4.0
Fish 2	23.9	1.2
Fish 3	73.9	5.4
Fish 4	72.3	1.7

### Dieldrin in mosquitoes and fish

Samples of adult male mosquitoes treated with dieldrin as eggs were analyzed and were found to contain an average concentration of 28.1 ± 2.9 µg/kg (mean ± standard deviation [SD]), with an RSD of 10.2%. The concentrations of dieldrin measured in the fish ranged from approximately 30 µg/kg to 74 µg/kg, with 3 of the 4 fish having concentrations above 65 µg/kg, (Table 3).

## Discussion

### Method development and validation

#### Mosquitoes

Based on a previous study by Pennell et al. [22], dieldrin concentrations in mosquito samples were first investigated using acetone and anhydrous sodium sulphate. With the amount of mosquito sample used (50 mg), this method did not produce satisfactory recovery yields. Orienting the method development process toward simplicity and efficiency, the use of salts was found to be superfluous, and acetone was the solvent yielding the best results—better than toluene and acetonitrile.

#### Fish

Based on several studies involving organochlorine residue analysis in fatty matrixes [18,23], a QuEChERS (Quick, Easy, Cheap, Effective, Rugged, Safe) methodology was further developed and adopted to determine the dieldrin in the treated fish. Some of the QuEChERS parameters needed to be adjusted to optimize the methodology. It was found that the extraction/partition step yielded better results with a water-to-acetonitrile ratio of 1:1, rather than using only acetonitrile.

The analytical sample size was investigated, and no significant discrepancies were found between using 1 g, 5 g, and 15 g. To reduce the time and chemicals used, the experiment was conducted using 1 g samples.

### Dieldrin residues in mosquitoes and fish

It is difficult to say whether the dieldrin levels found in the mosquito samples (28.1 ± 2.9 µg/kg) are generally high or low. In the 1980 s, Thome [11] found insects containing organochlorine pesticides such as dieldrin at levels ranging from 3 µg/kg to 70 µg/kg, which were levels considered to be relatively low for these insecticides at the time. Some samples even contained up to 274 µg/kg of dieldrin—a level considered to be high [11]. This level resulted from several environmental factors, although some of these insects were collected a considerable distance away from sites where the insecticide was sprayed.

The levels of dieldrin residues found per individual mosquito are very low (approximately 0.009 µg/mosquito). When several million treated mosquitoes are released into the environment, however, as would be the case in mosquito SIT operations using this mosquito strain, the environmental residue burden may become considerable, depending on the size of the treated area and the dispersion and dilution of the insects released. Using the values of the present experiment as an example, and assuming 1 million males are released per day over an area of 20 square kilometres, the area would be facing an environmental contamination of 452 µg dieldrin/km^2^/d, or 31 640 µg dieldrin/km^2^ in 10 wk. Control samples of soil, water, and fish or other natural predators of mosquitoes should be tested for the overall residue burden before and after releases to compare added residue levels in addition to background dieldrin levels, if any, as part of the pest control program's final evaluation.

Various risk assessment and management data, including tolerances or limits for dieldrin in water and in fish tissue have been published. The USEPA reports 96 h median lethal concentrations for dieldrin in water ranging from 1.1 µg/L for the susceptible rainbow trout to 41 µg/L for the most resistant goldfish [24]. Acute toxicity tests with aldrin and dieldrin have established that these compounds are toxic to freshwater aquatic life at low concentrations. The freshwater final acute value for dieldrin is 2.5 µg/L [24]. The Food and Agriculture Organization/World Health Organization's acceptable daily intake for the combined total of aldrin and dieldrin for humans is 0.1 µg/kg of body weight [25]. The US Food and Drug Administration has recommended an action level for aldrin/dieldrin of 100 µg/kg for the edible portion of fish [26]. There is currently no specific maximum residue level for dieldrin in fish in European legislation. To facilitate the control of residues of pesticides for which no maximum residue levels have been established, a default value at 10 µg/kg has been adopted [27]. Some countries in Europe—for example, The Netherlands and Germany—have set individual maximum residue levels for dieldrin and aldrin, expressed either singly or combined, in fish and fish products used for food, at levels of 200 µg/kg for fish and products made with fish, 100 µg/kg for eel, 200 µg/kg for fish liver, and 50 µg/kg for other fishery products [28].

A noncarcinogenic fish flesh criterion for piscivorous wildlife of 120 µg/kg has been developed [29], representing the concentration of dieldrin in fish above which wildlife may be affected. The dieldrin concentrations measured in the present study were for whole fish (with head removed) and not for the edible portions or for individual tissues, which may be more relevant for comparison with food tolerance levels. It should be noted that the fish species used was not a typical food for humans, and residues will vary in different species as well as in different tissues. Under the experimental conditions reported, however, our results clearly suggest that fish may accumulate dieldrin from feeding on contaminated mosquitoes to an extent where the concentrations incurred may be of concern if the fish were to be used for human consumption. The results found in the present study are similar (within a factor of 2) to maximum residue levels for fish consumption to protect human health. In the present study, the concentrations found were all below the US Food and Drug Administration action level of 100 µg/kg and below the fish flesh criterion for piscivorous wildlife. The variability of the results and the demonstrated bioaccumulation of dieldrin via dietary uptake, however, would indicate that it is possible that if the experiments had been carried on for longer, the action level could be exceeded. All fish tested in the present experiment were above the current European default maximum residue level of 10 µg/kg.

The results of the present study suggest that dieldrin is absorbed into the mosquito eggs during treatment and is retained until adulthood. These residues are then transferred up the food chain when ingested by natural predators, which subsequently bioaccumulate the residues over prolonged periods of time, even after the residue source in the diet is removed. In this case, using the approach described by Nebeker et al. [12] and assuming that a steady state (dieldrin concentration in fish tissue) has been reached by the end of the experiment, a bioaccumulation factor of 2.6 was estimated (calculated as the highest fish tissue dieldrin concentration [µg/kg] divided by the mean food [mosquito] dieldrin concentration [µg/kg]). These findings are consistent with those described by Nebeker et al. [12], in which tissue dieldrin concentrations were found to be 3.7 times higher than in the test diets in Mallard ducklings fed with dieldrin fed crickets, worms, or dieldrin-spiked commercial food, and with the bioaccumulation factor of 2.5 reported for Coho salmon feeding on dieldrin-contaminated Lake Ontario alewives and smelt [29]. Other studies involving controlled food chain transfer have reported bioaccumulation factors of 4.8, when feeding dieldrin-contaminated clams to blue crabs [9] or even a bioaccumulation factor of up to 1210 in the uptake of dieldrin from contaminated water by algae [10].

Amphibians, phyllopoda, insects, birds, osteichtyes, and marine mammals are said to be species with relatively high biomagnification factors for organochlorine compounds [30,14]. Fish were chosen for the present study because they are an important link in the food chain, are inexpensive, are easy to handle, eat mosquitoes readily, and have among the highest bioaccumulation rates for dieldrin [31] while still being resistant to its toxicity [24]. Fish retain dieldrin at higher levels from food sources than from water [31], and smaller fish have higher metabolic rates and may be able to excrete dieldrin residues at higher rates than larger fish with more body fat [31]. In terms of ingesting dieldrin-treated mosquitoes, the present experiment probably reflects a worst-case scenario for the hypothetical situation whereby the same fish ingests a large number of treated mosquitoes over a certain period of time. Conversely, however, being relatively small fish, the excretion of dieldrin may be higher than for larger, fattier fish. The results of the present study indicate that food-chain transfer of dieldrin residues from treated mosquitoes to fish is possible under our experimental conditions, which refutes the initial hypothesis that the residues retained by the adult mosquitoes would be negligible and would cause no concern with respect to the accumulation of the insecticide in the environment or the food chain. The results, however, cannot be used as a precise measurement of the quantity of dieldrin uptake by potential predators of the treated mosquitoes; but they can serve as an indicator that bioaccumulation of dieldrin residues is indeed an issue to be considered when using these insecticides for treating male *An. arabiensis* mosquitoes before release into the environment.

The present research is important in the context of mosquito AW–IPM control programmes that include a SIT component, in which the efficiency and practicality of the sexing process that requires using insecticides can be weighed against the potential environmental and health implications of releasing a large number of mosquitoes containing dieldrin (or other pesticides or chemicals) into the environment. With the currently available data, it is not possible to predict the pathological consequences for animals that feed on contaminated insects and for humans that consume these animals and thus experience an increased body burden of these compounds. These results confirm the need to reassess insect treatment protocols in the effort to minimize dieldrin concentrations and volumes used and to reduce insecticide waste. Follow-up experiments have been initiated in which different treatment protocols will be assessed regarding their effects on the mosquitoes' residue retention levels.

Every vector control strategy will have some impact on the environment. Aerial spraying of dieldrin and other persistent organochlorines in the 1960s and 1970s has left its mark, as residues from these pesticides still persist in the environment and in aquatic animals today [32]–[34]. The SIT is an attractive mosquito control tool, because it reduces dependence on insecticides. Therefore, taking into account the data presented in the present study, the development of a genetic sexing system for this mosquito species that does not require the use of insecticides is recommended.

## Conclusion

The present study describes an efficient, sensitive, and accurate method to detect dieldrin in exposed mosquitoes. The method was used to investigate the transfer of dieldrin from treated mosquito eggs via adult insects into fish fed on the mosquitoes.

Levels of dieldrin in the individual mosquitoes were found to be low (approximately 0.009 µg/mosquito) in the present study, and although actual contamination of the food chain cannot be predicted, the conflict between real and hypothetical risks may strongly impair decision-making in the potential implementation of the SIT component against this mosquito species. Fears arising from poor communication of such environmental issues could lead to a breakdown of public confidence in all mosquito SIT technologies, regardless of whether potentially toxic chemicals are involved or not. It is important that the environmental impacts and risks of using dieldrin-treated mosquitoes are evaluated for each individual project, site, and country as the risks can vary in severity depending on the geography, climate, culture, flora, and fauna. The degrees of acceptable risk also vary when weighed against the magnitude of the positive impacts of successful disease vector control.

Because dieldrin is so highly persistent and tends to bioaccumulate, the only way to reduce exposure to the general population and environment is to reduce its use. However, eliminating disease-transmitting female mosquitoes is of high priority, and a sexing system based on using an insecticide is an efficient and reliable method to achieve total sex separation. Therefore, there is a continued need to develop additional methods and strictly enforce protocols where the treatment of mosquitoes with dieldrin or other insecticides is necessary.
